# 

**Published:** 1860-03

**Authors:** 


### CONTENTS.

### MARCH, 1860.

### Lectures, Monographs, and Cases.

PAGE

Lectures on Displacements of the Uterus. By E. R. Peaslee, M.D., LL.D., &c. .... 577
The Power of Iodide of Potassium in Expediting Mercurial Salivation. By Bernard Kelly, M.D., &c. .... 586
Notes and Sketches, preparatory to a Treatise on Diseases of the Tropics. By G. Van Arcken, M.D., Bogotá, New Grenada .... 588
Uterine Polypus treated by Injections of the Perchloride of Iron. By J. N. Graham, M.D., Chicago, Ill. .... 599
Chloro-Anæmia treated with Bean of St. Ignatius. By Dr. Eisenmann, of Würzburg .... 601
The Effect of Solar Light on Vegetable and Animal Fæcula, &c. By Niepce de Saint Victor and Lucien Corvisart .... 602
Experimental Researches on the Action of Caustic Potassa. By Salmon and Maunoury, of Chartres .... 604
The Examination for Sugar in the Urine .... 605
Perchloride of Iron in Diphtheritis. By Dr. F. Isnard .... 610
Voltaic Narcotism .... 611
Arsenious Acid in Apoplectic Congestions. By Dr. Lamarc-Picquet, of Honfleur .... 612

### Monthly Summary of Medical Journalism,

By O. C. Gibbs, M.D., Frewsburg, N. Y.

A New Cure for Ingrowing Nail, 615; Asphyxia Neonatorum, 616; Treatment of Prolapsus of the Funis, 616; Gelsemin, 617; Lupulin in Delirium Tremens, 617; Chloroform and Cod-Liver Oil in Diphtheritis, 618; Puerperal Convulsions, 619; Bronchitis, 619; Belladonna as an Antigalactic, 620; Bronchocele, 620; Stomatitis Materna, 621; Inversion of the Uterus, 621; Miasmatic Hæmaturia, 621; Acetic Acid in Erysipelas, 623; Iodine Injections in Lymphatic Tumors, 623; Treatment of Indolent Ulcers, 623; Treatment of Intermittents, 623; Chlorate of Potash in Acute Rheumatism, 625; Cholera Infantum Cured by a Poisonous Dose of Fowler's Solution, 626; Hypophosphites in Phthisis, 626; Gastrotomy, 626; Iodide of Potassium in Diseases of the Brain in Children, 626; Ovariotomy, 628; Diphtheria .... 630

### Proceedings of Societies.

Annual Meeting of the New York State Medical Society .... 632
Academy of Medicine .... 644
Buffalo Medical Association .... 649

### Editorial and Miscellaneous.

Dr. Flint to the Subscribers of the *Buffalo Medical Journal*—Editors of the Monthly to its Subscribers—Dr. Green's Resignation—Meeting of the American Medical Association—Report of the New York Ophthalmic Hospital .... 652

